# A hope intervention for adolescents: a randomized controlled trial delivered by paraprofessionals

**DOI:** 10.3389/fpsyg.2025.1528504

**Published:** 2025-05-05

**Authors:** Anthony Scioli, Nathan MacPherson, Rachel Murphy, Tyler Gooding, Micah Love, Kathleen D. Lyons, Anna M. Adachi-Mejia

**Affiliations:** ^1^Keene State College, University System of New Hampshire, Keene, NH, United States; ^2^Geisel School of Medicine at Dartmouth, Hanover, NH, United States

**Keywords:** hope, clinical trial, emotion, adolescence, spirituality, group intervention

## Abstract

**Introduction:**

A workshop for adolescents was derived from an interdisciplinary model of hope. The workshop was created for delivery by professionals or lay helpers and is structured around the needs for attachment, survival, mastery, and spirituality.

**Methods:**

Adolescents, 13 to 17 years of age, received a five-week group intervention led by pairs of advanced psychology students. Hope, depression, anxiety, coping, and self-acceptance were assessed before and after the intervention. A delayed waitlist control group, matched for age, received identical outcome measures, also five-weeks apart. Group membership was randomly assigned.

**Results:**

More than three-quarters of the participants found the more left-brain writing exercises helpful, and nearly 85% rated the more right-brain reflections and meditative exercises favorably. Significant increases in hope as well as greater utilization of social coping methods and self-acceptance were found for the treatment group but not controls. Group participants (but not controls) also reported a significant reduction in depression. Anxiety levels were not impacted. Secondary analysis suggested that participant engagement and socioeconomic status may play a role in moderating the efficacy of this intervention.

**Discussion:**

This relatively low-cost intervention offers new hope for counteracting the global increase in youth despair. The effect sizes obtained in this study compare favorably with outcome data for cognitive-behavioral treatments and a few available agency-centered hope interventions. There is a potential for broad impact via the implementation of an accessible training program as well as online deliveries in either synchronous or asynchronous modes.

## Introduction

Adolescence is a critical juncture in the lifecycle, a time of accelerated physical, emotional, and social development. These changes can be stressful, leading to upheavals in perceived control, relationships, and self-regulation, as well as questions about identity and purpose (Holder and Blaustein, 2014; Petito and Cummins, 2000; McAdams and McLean, 2013). For these reasons, adolescence is also a time when significant mental health problems may emerge.

### Adolescent depression and suicide

While full-blown depressive states are uncommon in pre-pubescent children, rates of this disorder rise sharply in the teen years. In adolescence, the rate of depression is three to five times higher as compared to the childhood years (NIMH, 2014b) and nearly doubles between the ages of 12 and 13 (NIMH, 2014a). In 2015, more than three million teens between 12 and 17 reported at least one episode of major depression. In the same year, suicide was the third leading cause of death for individuals 10 to 14 years of age (NIH, 2015).

### Common factors in depression and suicide

At the psychological level, depression and suicide have been associated with disturbances in four domains of functioning: *perceived control, attachment, self-regulation, and existential-spiritual concerns.*

#### Perceived control and mastery

Depression can follow from experiences of helplessness and lack of control (Abramson et al., 1978). Weisz et al. (2001) compared factors related to depressive symptoms in children and teens and found that lack of perceived competence was the strongest predictor of depression in the older group. Quiroga et al. (2013) reported that self-perceptions of academic competence mediated the relationship between depressive symptoms in the 7th grade and eventual school dropout. Li et al. (2016) studied more than 1,500 Chinese adolescents. Suicidal ideation and attempts were correlated with over-control by parents. Auerbach et al. (2015) compared depressed adolescent suicide ideators and attempters. Attempters were more likely to demonstrate disrupted goal behavior, opting out of higher value but more effortful tasks in the face of uncertainty.

#### Attachment

Data from a National Institute of Child Health and Human Development (NICHD) study demonstrated that an increase in loneliness reported in middle childhood is predictive of depression in adolescence (Jones et al., 2011). Kouros and Garber (2014) reviewed evidence that inconsistent or harsh discipline, parental over-control, higher levels of family conflict, and lower levels of family cohesion may foster higher levels of adolescent depressive symptoms. Horton et al. (2016) identified feelings of thwarted belonging and perceived burdensomeness in a clinical sample of adolescents with suicidal ideation. Venta et al. (2017) found that low maternal trust moderated the relationship between depressive symptoms and suicide attempts.

#### Self-regulation

Compas et al. (2004) linked temperamental factors, including autonomic reactivity, to the generation of adolescent depression. In middle adolescents, an inability to handle negative emotions and to express positive emotions was predictive of depression concurrently and longitudinally (Caprara et al., 2010). Cairns et al. (2014) reviewed 113 studies on adolescent depression and discovered several self-regulatory risk factors, including negative coping strategies, poor diet, and decreased sleep. A review of completed adolescent suicides by Hoberman (1989) revealed deficits in affect and impulse management as well as poor problem-solving skills.

#### Spiritual and existential beliefs

Spiritual beliefs, religious involvement, or perceived support from a religious or spiritual community, may have a buffering effect on mental health, and may result in fewer symptoms of depression (e.g., Kim and Esquivel, 2011; Barton et al., 2013). Dew et al. (2010) controlled for initial depression, perceived social support and substance use in 145 adolescents, 12–18 years of age, and found that loss of faith remained an important predictor of depressive symptoms after 6 months. Reynolds et al. (2014) studied teens with diabetes or cystic fibrosis. In both groups, positive spiritual coping predicted fewer symptoms of depression over time. Among a sample of more than 1,000 Malaysian adolescents, those high in hopelessness and depression who reported being more spiritual, exhibited less suicidal behavior than those low in spirituality (Talib and Abdollahi, 2017). In Jewish adolescents, higher levels of religiosity are associated with fewer self-injurious thoughts and behaviors (Amit et al., 2014).

### Beyond CBT: improving interventions for diseases of despair in youth

For more than a half-century, investigators have pointed to hopelessness as a critical factor in the development of depression and the emergence of suicidal tendencies (Abramson et al., 1990; Franklin et al., 2017; Kazdin et al., 1986; Menninger, 1959). Hawton et al. (2013) reviewed 28 publications dealing with suicide and found that hopelessness doubled the odds of a self-induced death. Some progress is acknowledged in treating adult hopelessness using CBT-oriented programs (cognitive-behavioral therapy). For example, Gallagher et al. (2020) reported a moderate effect size of 0.65 from baseline to post-treatment for changes in hope, defined in terms of Snyder et al.’s (1991) “wills and ways” goal-expectancy model. Jiang et al. (2018) reviewed a Making Hope Happen (MHH) intervention as well as a “Building Hope for the Future” program (BHF), both based on the Snyder et al. model. While “significant increases” in self-reported cognitive-agentic hope were suggested, no effect sizes were provided. A separate search for data on each intervention revealed only two empirical studies. Buchanan (2008) implemented the MHH program with a sample of special education students and found no significant differences in hope scores, pre-to post-. Marques et al. (2011) reported an effect size of 0.92 in one BHF trial. Notwithstanding the most promising assumptions about these programs, the findings only provide support for a protocol that addresses a narrower conception of hope in terms of agency expectations. It is unclear whether changes in this reduced hope can produce downstream changes in youth depression, anxiety, or other derivatives of hopelessness.

Beyond hope-based protocols, reviews of the adolescent treatment literature reveal modest effects, including interventions based on CBT, with a mean effect size of 0.46 (Weisz et al., 2017). The data on treatment of youth depression, a disorder closely related to hope, shows even smaller effects. Weisz et al. found a mean effect size of 0.29 for all treatments of youth depression. To complicate matters, a review by Ng et al. (2020) revealed that only 83% of “candidate mediators” (potentially contributing factors), embedded within the youth psychotherapy literature, including CBT studies, were subjected to analysis, including treatment alliance, family functioning, group membership (attachment), or problem-solving shifts (coping/survival).

In summary, there are few youth interventions targeting hopelessness and overall, the effect sizes for youth psychotherapy are quite modest. There is a limited database of intervention studies highlighting the cognitive, goal expectancy model of Snyder et al. (1991). Taken together, these modest findings are not surprising, given that a greater constellation of factors are implicated in youth despair beyond agency or mastery, including attachment disruptions (Stoddard et al., 2011), hormonal-related coping/emotional dysregulation challenges (Ho et al., 2022), and emerging spiritual individuation (Miller and Barton, 2015).

A broader approach to hope is needed to generate more effective treatments for despair in youth. The predominant psychological approaches to hope and hopelessness have emphasized a cognitive-goal-expectancy perspective (e.g., Gallagher et al., 2020; Mowrer, 1960; Stotland, 1969; Snyder et al., 1991). Going further, cognitive-behavioral approaches to hope are rooted in Western concepts of mastery (perceived control) and an emphasis on personal agency (Mowrer, 1960; Snyder et al., 1991; Stotland, 1969). These goal-oriented approaches overshadow classic psychodynamic writings on the topic which highlight the importance of trust, connection, and mergers with powerful others (Erikson, 1950; Kohut, 1971; Pruyser, 1986) as well as the work of clinical psychologists and stress researchers on the self-regulatory aspects of hope (Breznitz, 1986; Folkman, 2010). Beyond psychology, theologians and philosophers associate hope with transcendent motives and perceived sacred relationships (May, 1975; Marcel, 1962; Moltmann, 1993; Tillich, 1952).

### Fundamental hope: hopefulness

The literature on hope encompasses both processes (hoping) and character (hopefulness). In the psychodynamic and philosophical literature, the latter is emphasized and labeled “basic” or “fundamental” hope (Erikson, 1950; Marcel, 1962). Nursing and psychology tend to focus on the former, what Marcel (1962) labeled “ultimate hope” (e.g., a hope for recovery or career achievement). The research on depression and suicide points to a combination of trait and state factors (Daniel et al., 2017; Klonsky and May, 2015). In short, we can presume that disruptions in either fundamental or ultimate hope may compromise health. *Nevertheless, for philosophical and practical reasons, the focus of our lab, and the research we describe in this study, is on basic or fundamental hope.*

We presume that fundamental hope develops before the appearance of ultimate hopes. Fundamental hope is more likely to precipitate, or influence waxing and waning ultimate hopes. Moreover, even in the presence of extraordinary life changes, dispositional factors such as fundamental hope may affect the nature of emergent ultimate hopes (Scioli and Biller, 2009). Fundamental hope is an intriguing clinical target for preventive and developmentally focused research. For example, Larsen and Stege’s (2012) review of hope-related themes in counseling sessions suggest that goal attainment is typically a minor theme, eclipsed by client-therapist relationship building, identity development, and perspective change. Dubois and Beauvois (2005) found that self-sufficiency, self-anchoring, and internality, but not goals, rated high in desirability and utility, even in non-collectivist nations.

Scioli and Biller (2009, p. 30) define fundamental hope in the following manner:

“Fundamental hope is a future-directed, four-channel emotion network … The four constituent channels involve the attachment, survival, mastery, and spiritual systems (or sub-networks). The hope network is designed to regulate these systems via both feed-forward (expansion) and feedback (maintenance) processes that generate a perceived probability of adequate presence and power as well as protection and liberation”.

**Figure 1:**
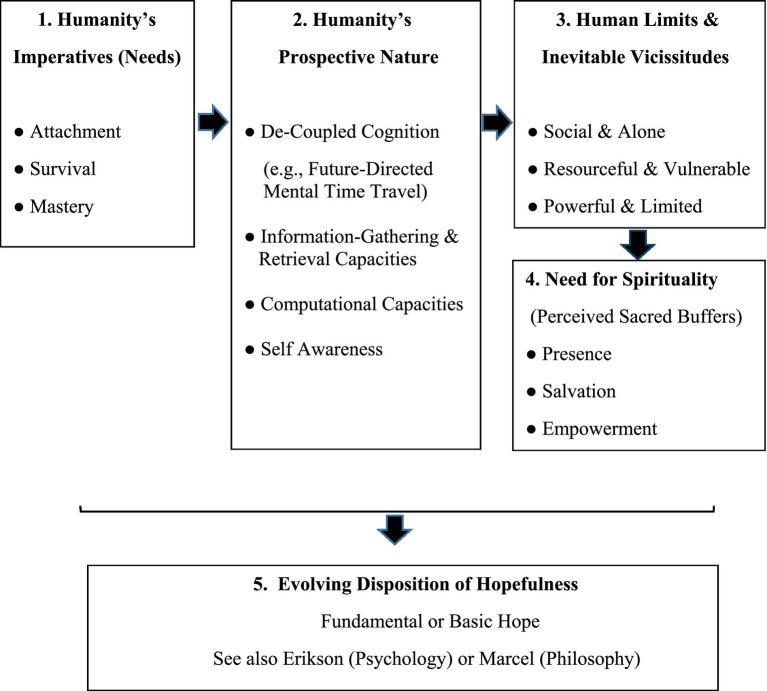
A chronological derivation of fundamental hope (trait hope or hopefulness).

Fundamental hope derives from basic needs as well as humanity’s capacities for memory, likelihood estimations, and prospective thinking (Rychlak, 1993; Seligman et al., 2016), in interaction with the inevitable vicissitudes of life (see Figure 1). These factors give rise to four dimensions of hopefulness. The attachment dimension of hopefulness encompasses trust, openness, and perceived connectedness (Erikson, 1950; Marcel, 1962). The survival aspect features liberation beliefs and self-regulative reality-negotiation strategies that include image-building (reality-construction) and evidence-gathering (reality surveillance) (Breznitz, 1986; Pruyser, 1986; Wright and Shontz, 1968). The mastery component reflects higher goals and ideals, empowerment beliefs, and collaborative action tendencies (Emmons, 1999; Lynch, 1965). The spiritual dimension is a by-product of inevitable shortcomings in addressing the attachment, mastery, and survival meta needs. Individual and cultural factors determine the level of investment in spiritually based experiences of presence, salvation, and/or empowerment.

#### Instilling fundamental hope: a whole-brain approach

Scioli et al. (2011) introduced a five-phase intervention targeting four dimensions of fundamental hope (see uploaded Supplementary file for further details). Two modules are devoted to attachment. The remaining three modules address mastery, survival/coping, and spirituality. Each module includes three types of activities: reflections and discussions (classic wisdom quotes); writing experiences (integrative psychosocial skill-builders); meditative–hypnotic exercises. An R-A-P method was used to facilitate discussion of the classic quotes (hope wisdom). What is the *real* meaning of the quote (larger message)? Why should we *appreciate* this idea (e.g., expert opinion, recognized genius, passed test of time)? What is the *proof* or evidence for this idea (e.g., logic, emotion, or data, i.e., a rational, mystic, or empirical way of knowing)? (Note: We also emphasize that “RAP” defines a style of music or frank speech and is a homonym of the word “wrap,” suggestive of the English idiom of “wrapping one’s mind around an idea.”) The skill-building exercises reflect cognitive-behavioral, humanistic, dynamic, or existential concepts as well as ideas culled from attachment theory. The concluding meditative practice consists of two parts, imagery to induce whole body relaxation (the meditative “vehicle”), and suggestions (the “intervention”) to reinforce hope lessons of the week (e.g., attachment hope or mastery hope).

The combination of techniques addresses the whole brain. Reflections on quotations, particularly philosophical concepts and metaphors impact the right and left-brain. The integrative exercises primarily engage the analytic left–brain. The meditative practices primarily engage the right brain but also the left–brain. More broadly, philosophical reflections and classic quotations establish mindsets and new ways of seeing the self, the world, and the future. Wittgenstein (1953/2009) described philosophy as a way of being and a way of seeing, touting the power of holding “pictures in the mind.” Research by Wiitala and Dansereau (2004) highlights the value of adding quotations to writing exercises to counter “closed loop thinking.” New ideas (mental pictures) break into a closed loop and generate fresh possibilities. The psychosocial exercises instill skillsets. The emphasis on writing (longhand) during both phases (quotes and skill builders) incorporates research on its role in deepening understanding and increasing retention and recall. Moreover, there is evidence that longhand writing engages the motor and sensorimotor areas of the brain (James, 2017; Mueller and Oppenheimer, 2014), increasing links between thought and future action tendencies. The meditation exercises consolidate hope lessons and instill hopefulness as a way of being.

The philosophical reflections (on quotations) include affirmations and life-lessons offered by thinkers from every age and wisdom tradition (e.g., Aristotle, Henry Ward Beecher, the Dakota and Seneca Indians, Yiddish proverbs, Ecclesiastes, the Koran, Buddhist teachings, Gandhi, and Tolkien). Four examples follow, from the attachment, survival, mastery, and spiritual modules.

“There are only two lasting bequests we can hope to give our children. One of these is roots, the other, wings."—Henry Ward Beecher (Attributed, or Hodding Carter, 1953, p. 337)

“Life is like a game of cards. The hand that is dealt to you is chosen for you; the way you play it is chosen by you.”—Jawaharlal Nehru (Attributed)

“I was on the bank of a river … One of my teachers was on the side I was trying to reach … my mother and father were on my side. They started out with me … They went as far as they could go and mother turned and said to me—“My child, we have brought you as far as we can go … we must leave you and you must make it for yourself.”—Mary McLeod Bethune (in McCluskey and Smith, 2002).

“In the Middle Ages, a favorite image is the wheel of fortune … if you are attached to the rim of the wheel of fortune, you will be either above, going down, or at the bottom, coming up. But if you are at the hub, you are in the same place all the time … centered.”—Joseph Campbell (Campbell, 1991, p. 147)

Research on affirmations demonstrates their value in increasing self-awareness, implementing associated behaviors, fostering self-control, and regulating stress (Burson et al., 2012; Ferrer et al., 2012; Harris, 2011; Sherman, 2013). Encouraging philosophical reflections in teens is a more recent development. A successful program of using philosophy with 10 and 11-year-old pre-adolescents and aided by undergraduates, was conducted by Kaye and Thomson (2006) via the Carroll-Cleveland Philosophers Program. Cognitive-behavioral, humanistic, and meditative approaches are standard in psychotherapeutic practice with adolescents (Beck et al., 2012; Jayne and Ray, 2016; Thurman and Torsney, 2014; Webb et al., 2012). Existential approaches to treating adolescents are less common. Shumaker (2012) lists a variety of development factors in adolescence that portend a broader and deeper set of existential challenges, including: increased meta-cognitive abilities, greater introspection, awareness of contradictory elements of reality, reflections on matters of authenticity, qualitative changes in identity formation, and a fuller realization of one’s mortality.

### A hope-centered workshop

The hope-centered workshop represents a theoretically integrated intervention (Norcross and Goldfried, 2005). Integration occurs both within modules (e.g., existential and dynamic exercises) and within individual exercises (e.g., CBT and humanistic elements).

The *hope chest* exercise in the attachment module offers a good example of integration. Teens receive scrolls for compiling a list of important people and possessions after considering specific criteria (safety and trustworthiness). This exercise borrows from Erikson’s psychosocial focus on basic trust, Joiner’s (2002) interpersonal theory of suicide, the writings of Rogers (1953, 1985) on acceptance and positive regard, and attachment theory (Bowlby, 1969).

The survival module targets perceived degrees of freedom (liberation beliefs) and ways of coping. A *stretch-your-potential* exercise draws on the writings of existential psychologist Rollo May (1975). Teens reflect on four “facts of life” (time and place of birth, genetics, culture, and unpredictable world events) as sources of strengths rather than limiting factors. This exercise also reflects creativity and coping research on the value of “binocular” perceptual processes (combined positive–negative information-processing) (Bion, 2004; Wright and Shontz, 1968; Folkman, 2010).

The *options-for-coping* exercise derives from the cognitive-behavioral formulations of Folkman and Lazarus (1988). Workshop leaders explain how spiders may rely on seven different types of webs to deal with diverse environmental challenges. Participants learn eight ways of coping and five ways of scanning their internal and external environment. They also evaluate the benefit and/or harm of each strategy in the context of a bullying scenario.

The mastery module incorporates the social and existential research on values by Rokeach (1973) and Kohut’s (1971) psychodynamic understanding of empowerment. A *success formula* is defined (values + strengths + role models + commitment = success). Selected Rokeach values highlight attachment, survival, and mastery commitments. Teens review valued outcomes in terms of strengths and weaknesses as well as role models and external obstacles.

The spiritual module enlarges the concept of spirituality to include nature, art, and science. A “spiritual type” questionnaire helps teens to identify whether they are an independent, follower, collaborator, mystic, or sufferer. This typology builds on the work of religious scholar Houston Smith (2006) and Fowler’s (1981) cognitive-developmental model of faith development. Teens evaluate their affinity for various nature, art, or science activities, in light of their spiritual types. They leave with a take-home handout on seven religions that includes tables for reading and music (left and right brain), divided by faith traditions, and six spiritual types.

The meditations that close each module begin with a standard progressive relaxation script (“waves of relaxation”), followed by concepts introduced during the week (module). Each module consists of two 45-min sessions. The entire workshop spans 5 weeks.

#### Previous pilot studies

The intervention was pilot tested four times, twice with youth in the US and Pakistan, a third time with early adolescents in Haiti and a fourth with middle adolescents in South Africa. A psychologist or career counselor led each group. Hope scores increased significantly from pre-to post-intervention, with an average effect size of 0.87. This magnitude of change compares favorably with those reported in meta–analysis of clinical trials of standard cognitive-behavioral therapy (CBT) or antidepressant drugs (TADS; Kratochvil et al., 2006; ADAPT: Wilkinson et al., 2009), as well as reviews by Klein et al. (2007) and Weisz et al. (2006). The qualitative feedback was positive…“This was a life changing experience” … “I started with a demoralizing mindset [but now] my mindset is very different and positive”… “The activities and exercise were interactive and well planned”… “This group helped me to understand my purpose”…“Extremely inspiring”… “I now want to be a messenger of hope for others.”

### Focus of the present research: feasibility and efficacy

The pilot studies focused on a single outcome (hope) and did not include a control group. The leaders were professionals. In the present research, we added random assignment, a control group, a broader set of outcome targets, and utilized trained undergraduates to deliver the intervention (“hope providers”). The focus in year one was on feasibility, and in year two, efficacy.

## Materials and methods for study 1: feasibility

In year one, the aim was to train students in a multidimensional model of hope, adolescent development, and the basics of effective workshop delivery. *We anticipated that young adult paraprofessionals could assimilate an integrative theory of hope and engage with a translation of this model into an application.*

### Hope provider training

The training team consisted of a clinical psychologist with expertise in hope and two other collaborators from the Geisel School of Medicine at Dartmouth (an adolescent health research expert and an occupational therapist with expertise in conducting clinical trials). The hope providers were three male and one female undergraduate psychology students ranging in age from 20 to 26 (M = 21.75, SD = 2.87). We selected hope providers using a two-step process; faculty nominations, followed by individual interviews. We asked students for a three-year commitment.

We began training with 4 months of instruction in hope theory and the nature of adolescence (readings, written annotated summaries, discussion). We followed with 4 months on workshop delivery (reading, discussion, videotape role-plays, and focus groups with a mock set of participants). We ended year one with 4 months of training and practice on advanced statistical procedures and data analysis. To evaluate theoretical assimilation, the principal investigator (PI) read students’ written summaries of each training module and led follow-up discussions. To evaluate fidelity of delivery, the PI evaluated presentation videotapes and led a focus group with participants in the mock intervention (additional undergraduate volunteers). In year two, the hope providers delivered the intervention and received 90 min of supervision, twice a week (60 min before each session; 30 min after each session). The focus in year three was on data analysis, conference presentations, and manuscript development.

There are historical, practical, and theoretical reasons for advancing a multi-tiered approach to instilling hope (faculty to undergraduates to teens). Many psychology undergraduates aspire to work in an applied setting, pursue related coursework (e.g., counseling techniques), and even practicums. Secondly, the hope intervention is highly structured (manualized), making it accessible to lay providers. Thirdly, there is theoretical justification for a multi-generation distribution of hope provided by the work of Russian psychologist Lev Vygotsky (1978) via his concept: the “zone of proximal development.” Faculty and other senior professionals can lead undergraduates through one proximal zone of hope-related growth. Descending one level on the developmental ladder, college students can lead adolescents through a more basic zone of hope-related growth. Adolescents may view undergraduates as embodiments of an upcoming phase of human development, what they can hope for in terms of attachment, mastery, survival, and spirituality in their “next life.” Successful examples of pairing undergraduate mentors with adolescent learners include the Ed. Portal Mentor Program (Harvard University, 2018) (mastery focus), the College and High-School Alliance Mentoring Program (University of the Redlands, 2016) (attachment and coping skills), and the Vision Youth Academy (University of Notre Dame, 2018) (spirituality).

## Results study 1: feasibility

### Theoretical assimilation

The students assimilated the hope model and related literature on adolescence with little difficulty. Two concepts required a more intensive review. Students (and the lay public) are more apt to focus on the verb form of hope (hoping). After some additional discussion, students became better versed in the concept of fundamental hope (hopefulness). The nature of spirituality also required additional discussion. For many students, spirituality equals “religion” or “god.” We stressed a broader, relational definition (self in relation to a sacred object or endeavor).

### Application

All student leaders had experience working with children or teens. One was a volunteer for a Big Brothers–Big Sisters organization. Another had worked as a summer camp counselor. Two students had assisted in running therapeutic groups for teens. Careful selection of hope providers, coupled with previous experience, and ongoing supervision, resulted in very few challenges with respect to workshop delivery. The two areas that required some supervisory inputs were “boundaries” and “limit setting” (e.g., teen requests for social media contact with providers or acting-out behaviors during a workshop session).

We asked each pair of leaders to independently rate participants’ level of engagement in the group (1 = disengaged, 2 = weakly engaged, 3 = engaged, 4 = strongly engaged, 5 = very strongly engaged). The inter-rater reliability was 0.85. The mean engagement score was 3.60 (SD = 1.06). Leaders rated 27 of 31 participants (87.09%) as engaged, strongly engaged, or very strongly engaged.

We collected open-ended feedback responses from 30 of the 31 treatment participants (96.7%). Participants received simple instructions: “Use this blank form to give us some feedback about your experience in this group.” Two raters sorted the responses into nine categories. We summarize these responses in Table 1. More than 80% of the participants reported a positive emotional experience. Among those who highlighted learning, 70% mentioned new insights into the nature of factors that can generate hope. Among those who commented on the leaders, nearly 90% expressed a positive view. Participants also wrote favorably about the meditation exercises (83% positive). While fewer in number, references to student interaction, spirituality, and the introductory videos were more positive than negative. We received mixed feedback on the writing activities. The primary negative response was that they were challenging and required effort. One student reported a desire for a more relaxing physical environment.

**Table 1:** Participant feedback.

Themes	Total references	Positive comments	Negative comments	Neutral comments
Overall emotional experience	17	14	3	0
Writing activities	11	4	5	2
Learning/insight	10	7	3	0
Meditation	12	10	2	0
Spirituality	3	2	1	0
Quality of student interaction	4	3	1	0
Quality of leaders	7	6	1	0
Quality of videos	2	2	0	0
Room quality	1	0	1	0

## Materials and methods for study 2: efficacy

The focus in year two was to assess the efficacy of the hope-centered workshop, specifically for adolescents with mild to moderate depression, delivered by young adult hope providers. *We hypothesized that the treatment group but not the control group would demonstrate a significant increase in hope and self-acceptance, greater use of social coping strategies, as well as decreases in depression and anxiety.*

### Study design

**Figure 2:**
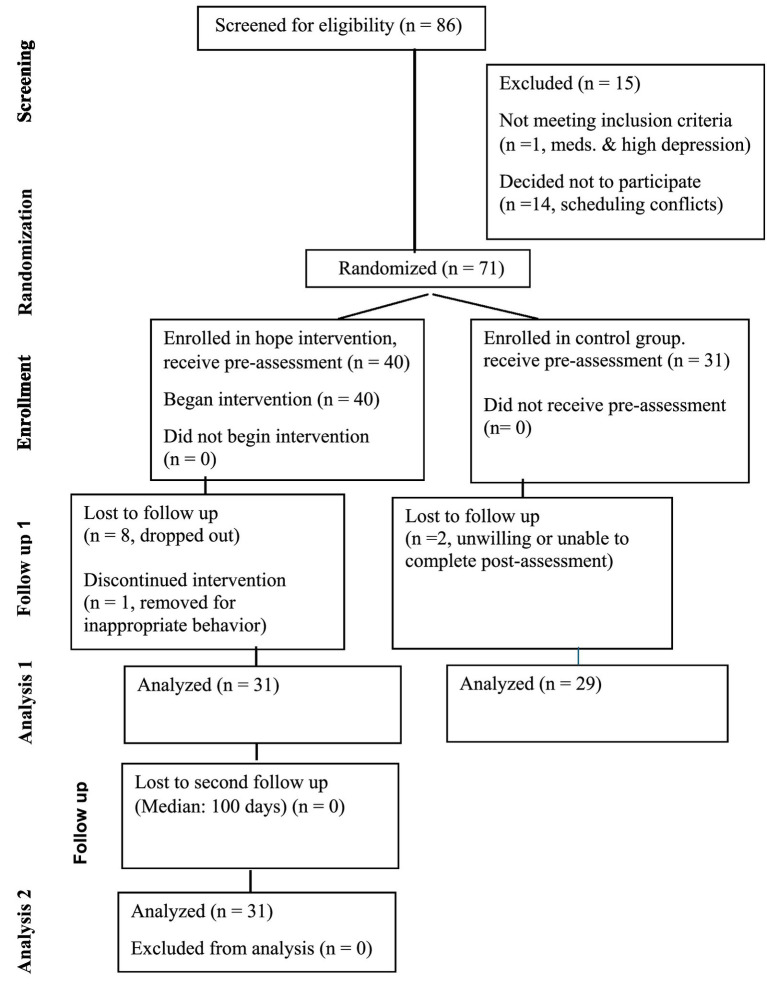
CONSORT diagram for flow of participants through each stage of randomized trial.

We followed a randomized treatment and delayed-waitlist control design. We administered a pre-assessment battery to both groups. A five-week intervention was provided to the treatment group. Subsequently, we administered a post-assessment to both groups (See Figure 2). Three months after the post-test we assessed hope again in the treatment group.

### Participants

Following study approval from the IRB (Institutional Review Board for ethical research), we recruited 86 participants from local and regional mental health centers as well as social media and newspapers. We described the group experience as a character-building intervention rather than formal therapy. If a participant demonstrated a worsening of depressive symptoms or the emergence of suicidal thoughts, the PI would contact the participant’s mental health provider or primary care physician. This exception to confidentiality was included in the consent form.

#### Eligibility criteria

We collected screening and outcome data online using Qualtrics. Participants provided an email address and received instructions for logging in with a confidential ID. We excluded teens with Child Depression Inventory (CDI) scores above 37 (severe) or who indicated, “I want to kill myself” (CDI Item 9, Score 2). We also excluded teens currently prescribed antipsychotic medications. The inclusion criteria included age (13 to 17 years), participant assent, and parental consent. One student was excluded and referred to his parents and healthcare provider due to medication dependence, severity of depression, and expressed suicidal desire. Another 14 students opted out of the treatment before assignment due to foreseeable scheduling conflicts. We randomly assigned 40 of the remaining 71 teens to an intervention group, and 31 to a wait-list control group. Participants received 10.00, 15.00, 20.00, and 25.00 gift cards for completing screenings, pre-group assessments, post-group assessments, and follow-up assessments.

### Delivery of the workshop intervention

We conducted the workshops on Tuesdays and Thursdays for 5 weeks. The standard configuration was two hope providers (male and female) and six teen participants. Each meeting lasted 45 to 60 min and took place in the afternoon. On Tuesdays, leaders began with a short, 3 to 5-min video highlighting the hope theme of the week (e.g., attachment or survival). A writing and discussion session followed, using hope quotes selected for philosophical and metaphorical content (using the R-A-P method presented in a Socratic style). Leaders introduced integrative life-skills writing and discussion exercises in the second half of each Tuesday session, which continued through the first half of each Thursday session. The second half of every Thursday was set aside for a meditative-hypnotic exercise. Unlike hypnosis, we did not introduce a trance component. Relaxation imagery was followed by a hope theme suggestion. In total, we ran five groups.

### Outcome measures

#### Fundamental hope

The Comprehensive Child and Adolescent Hope Scale (CCAHS; Scioli et al., 2016) was used to measure hope before and after the group. The CCAHS is 40 item, Likert-styled questionnaire (never = 0, sometimes =1, always = 2) with items covering the four dimensions of hope (attachment, survival, mastery, and spirituality). The questionnaire is internally reliable (Alpha = 0.95) and stable (three-month test–retest = 0.77). Hope total and sub scores correlate with theoretically linked constructs, including positive aspects of the self-concept (Piers-Harris Scales (PH); Piers and Herzberg, 2002). For example, PH Intellectual Self-Perception correlates with CCAHS Mastery Hope (r = 0.79); PH Popularity correlates with Attachment Hope (r = 0.64); and PH Freedom from Anxiety correlates with Survival Hope (r = 0.58) (all *p* < 0.05). Higher hope scores also correlate with parental reports of less anxiety and depression on the widely used Child-Behavior Checklist (CBCL; Achenbach and Rescorla, 2000).

#### Depression

The Child Depression Inventory (CDI; Kovacs, 1985) is the most widely used measure of depressive symptomatology for children and adolescents ages 7 to 17. Internal consistency reliability values are strong (alpha values = 0.80 to 88). The CDI has good face validity, covering 8 DSM depression categories. CDI scores correlate with measures of anxiety and self-esteem in expected directions.

#### Anxiety

We assessed anxiety with the short form of the revised Children’s Manifest Anxiety Scale (RCMAS II; Reynolds and Richards, 2008). The RCMAS II short form is a ten-item, yes-no formatted questionnaire to measure anxiety in children 6 to 19 years. Factor analysis has confirmed a one-factor structure (Lowe, 2015). RCMAS scores correlate with other standard measures of anxiety and in one study, higher scores were predictive of viewing ambiguous stimuli as threatening. The short form provides a measure of chronic, manifest anxiety. In the present sample, the alpha value was 0.76.

#### Coping

We created a social coping measure by combining three items from the Adolescent Coping Orientation for Problem Experiences (ACOPE; Patterson and McCubbin, 1987) with two new items. The ACOPE is a self-report, Likert-style questionnaire for adolescents to indicate their use of specific coping behaviors (Never (1) to Most of the time (5)). There are multiple subscales including humor, avoidance, and social support. Scale Alpha values range from 0.06 to 0.75. (Mean = 0.53). We created a more theoretically relevant coping scale entitled “hope provider recruitment” by summing the five items (below). The alpha for this scale was 0.59.

1. Talk to a friend about how you feel (ACOPE 52): Attachment.
2. Be with a boyfriend or girlfriend (ACOPE 16): Attachment.
3. Let off steam by complaining to friends (ACOPE 22): Attachment-Self-Regulation.
4. Reach out to a good problem solver (New): Attachment-Survival.
5. Imagine what a hero or role model would do (New): Spiritual-Inspiration.

#### Self-acceptance

The Ryff and Keyes (1995) measure of Self-Acceptance is a subscale from their omnibus measure of subjective wellbeing (SWB). The measure consists of nine items, scored via a Likert Scale (1 = strong disagreement to 6 = strong agreement) (Alpha = 0.93). Self-acceptance scores correlate with measures of positive affect, life satisfaction, and lower depression (Ryff and Keyes, 1995) as well as extraversion, agreeableness, and conscientiousness (Schmutte and Ryff, 1997). We selected **s**elf-acceptance as our measure of wellbeing for several reasons; empirically, it is the SWB scale with the strongest inverse correlation with depression and has the highest positive correlation with positive affect and life satisfaction (Ryff and Keyes, 1995). Moreover, self-acceptance is critical in the teen years and may serve as a buffer in individuals who self-identity as socially disadvantaged in one or more domains (e.g., status or popularity) (McElhaney et al., 2008).

## Results: study 2: efficacy

### Preliminary analysis 1: retention and comparability of study groups

Within the control group, 31 of the 40 teens were retained through completion of the study. One student was removed due to inappropriate behavior. Another 8 students dropped out, citing unanticipated scheduling conflicts, family relocation, or “lack of interest.” A comparison between the controls and dropouts should not be overinterpreted given the small number of the latter. However, the participants who dropped out did report lower initial (pre-) hope scores (t (34) = 2.58, *p* < 0.05). There were no significant differences in age, initial depression scores or family SES levels (all *p* > 0.05).

The ages were comparable for intervention and wait-list controls (Treatment age = 15.39, SD = 1.06; Control age = 15.88, SD = 1.04; t (58) = 1.78, *p* > 0.05). A higher percentage of males enrolled as controls (Treatment: males 11/31 (35%), females 20/31 (65%); Controls: males 14/27 (52%), females: 13/27 (48%)). (Two control participants reported “other” for gender). The difference in proportions was not significant (X^2^ (2) = 3.78, *p* > 0.05).

### Preliminary analysis 2: pre-intervention correlates of fundamental hope

Within the intervention group, neither age nor family SES were correlated with hope (r = 0.17 and 0.16, respectively, *p* > 0.05). Male and female hope scores were comparable at baseline (Males = 79.97, SD = 6.11; Females = 80.49, SD = 7.64; t (29) = 0.20, *p* = 0.85). We also obtained pre-intervention data regarding grades and academic effort (items from the Youth Risk Behavior Survey (YRBS; Centers for Disease Control and Prevention, 2016)). Using a more goal–oriented measure of hope, Snyder et al. (1991) found that hope correlated with college achievement. We asked the following questions. During the past year, how would you describe your grades in school? The response choices range from A (Mostly A’s) to E. (Mostly F’s). During the past year, how much effort did you make in school to do your best? The response choices range from A (100 percent) to F (10 Percent). Hope scores were positively related to both self-reported grades and academic effort (r = 0.43, *p* = 0.04; r = 0.45, *p* = 0.03, respectively).

### Primary analysis: assessment of five outcomes

We conducted a series of mixed ANOVA’s (condition × time) for each of the five outcomes. We could not evenly divide the samples in either group by middle or late adolescence, and instead inserted age as a covariate. For gender, we excluded the two non-binary participants. Three of the five interactions were significant (Hope, *F* (1,57) = 7.25, *p* = 0.01; Depression, *F* (1, 57) = 5.88, *p* = 0.02; Self-Acceptance, F (1,57) = 4.09, *p* = 0.04). For Anxiety and Coping, the interactions were not significant (Anxiety, F (1,57) = 0.76, *p* = 0.39; Coping, *F* (1,54) = 0.09, *p* = 0.76). The simple effects tests appear in Table 2. Hope and Self-Acceptance increased significantly in the treatment group but not the control group. Depression showed a significant decrease in the treatment group but not the control group. For anxiety and coping, we followed the recommendations of Wei et al. (2012) for assessing the means of non-interacting factors by successive examination of all potential differences across conditions and time to confirm only a simple effect. As shown in Table 2, for anxiety, neither group showed a significant change over time. However, the coping simple effect was significant for the treatment group but not the control condition.

**Table 2:** Intervention impact on hope, depression, anxiety, coping, and self-acceptance.

		Pre-TX		Post-TX		Change	T	*p*	Effect Size
	N	M	SD	M	SD				
Treatment groups									
Hope (Total)	31	80.31	7.03	83.71	8.35	3.40	3.56	0.01	0.64
Depression	31	15.59	8.87	12.67	6.47	2.92	2.43	0.02	0.44
Anxiety	31	5.03	2.56	5.10	2.55	0.07	0.35	0.73	0.06
Coping	31	12.89	3.38	14.08	3.11	1.19	2.53	0.03	0.45
Self-acceptance	31	24.85	6.17	28.03	6.07	3.18	3.02	0.01	0.54
Control groups									
Hope	29	84.22	9.03	84.45	8.77	0.23	0.18	0.86	0.03
Depression	29	13.09	7.69	13.96	8.34	0.87	1.03	0.31	0.19
Anxiety	29	4.53	2.84	4.19	2.91	0.34	1.08	0.29	0.20
Coping	29	13.92	2.92	14.82	3.58	0.90	1.67	0.05	0.27
Self-acceptance	29	28.51	4.99	28.99	5.52	0.48	0.63	0.53	0.12

We also examined the outcomes within the treatment group for each of the four dimensions of hope. Significant increases were found for three of the four subscales (Attachment Hope, t (30) = 2.91, *p* < 0.01, es = 0.42; Survival Hope, t (30) = 4.92, *p* < 0.01, es = 0.91; Mastery Hope, t (30) = 2.42, *p* < 0.05, es = 0.44). Spiritual Hope trended upward (t (30) = 1.26, *p* = 0.22, es. 0.23).

To assess the *durability* of intervention effects, we assessed hope 3 months after treatment (median time = 100 days). The increases from baseline remained significant for Total Hope (t (30) = 2.12, *p* = 0.042, es = 0.38).

### Secondary analysis 1: SES and intervention engagement

We created low and high SES scores (median splits). The interaction for hope was nonsignificant (*F* (1,28) = 0.89, *p* = 0.36). An exploratory analysis revealed that both low and high SES participants reported a significant increase in hope (both *p* < 0.05; es = 0.59 for low SES, es =0.70 for high SES). The engagement x hope interaction was also nonsignificant (*F* (1, 28) = 0.04, *p* = 0.84). Hope increased significantly regardless of participant engagement level (less engaged: t (13) = 2.99, *p* < 0.05, es = 0.80; more engaged: t (16) = 2.23, *p* = < 0.05, es = 0.55).

### Secondary analysis 2: impact on 10 minor hope subscales

We compared effect sizes (pre to post change) across the 10 lower-order hope subscales. For Mastery Hope, there was a stronger effect for Supported Strivings vs. Ultimate Gains (perceived goal realization) (0.41 vs. 0.06). For Attachment Hope, the effect sizes were comparable for Trust, and Bonding (social connections) (0.26 and 0.30). For Survival Hope, Liberation Beliefs were most strongly impacted (0.67), followed by Interpersonal Assurance (0.45), then Self-Regulation (0.43). Spiritual Presence scores remained virtually the same (t (30) = 0.26, *p* = 0.79). Both Spiritual Assurance and Spiritual Empowerment trended upward (t (30) = 1.99, *p* = 0.056; t (30) = 1.67, *p* = 0.11). We created a composite “protection and empowerment” variable by summing Spiritual Assurance and Spiritual Inspiration (removing Spiritual Presence). The increase was significant (t (30) = 2.19, *p* = 0.04, es = 0.40).

## Discussion

This study provides empirical support for a hope-centered intervention based on an interdisciplinary integration of the literature (Scioli et al., 2011; Scioli and Biller, 2009). Due to its novelty, complexity, and reliance on paraprofessionals, we assessed feasibility as well as efficacy. College students with a strong orientation in fundamental hope (hopefulness) and basic training in workshop delivery can provide an engaging and effective intervention. We found significant shifts in four of five targeted outcomes, including increases in three positive outcomes (hope, self-acceptance, and social coping) and decreases in one negative outcome (depression).

### Feasibility

The feedback from adolescent participants was generally favorable. There were minimal disruptions. We cannot overestimate the importance of selecting appropriate hope providers (workshop leaders) with emotional intelligence and an appreciation of philosophy and literature (i.e., “wisdom culture”). We opted for an open-ended participant feedback form. We plan to convert participant responses into a Likert styled process questionnaire in future trials.

Over 80 percent of the teen participants reported a positive emotional experience. Seventy percent commented on “new learning” and nearly 90 percent of the comments about the leaders were positive. Some of the participants found the writing to be taxing, preferring more time for unstructured discussions. We plan to introduce minor changes in future groups to strengthen participants’ motivation for writing via an introductory message, a maxim on the value of writing, and a brief addition to each week’s meditation.

We examined several potential moderators, including engagement. Overall, nearly 90 percent of participants were rated as engaged, strongly engaged, or very strongly engaged. The effect size for hope rose from “moderate” (0.61) in highly engaged participants to “large” (0.80) in *lower* engaged participants. This suggests that the group process may instill hope even when engagement is less than optimal.

### Efficacy

#### Hope

Hope scores increased significantly. To assess the magnitude of change, we computed Cohen’s d, an uncorrected index of effect size. Cohen’s d for total hope was 0.65. For the subcomponents of hope, the values ranged from 0.23 (Spiritual Hope) to 0.91 (Survival Hope). For non-spiritual hope (sum of Attachment, Survival, and Mastery), the effect size was 0.77. The effect size was larger for participants rated as less engaged and who were from higher SES backgrounds. For hope, and the other outcome variables, we also computed Hedges g, a corrected effect size value (Hedges and Olkin, 1985). The uncorrected and corrected effect sizes were essentially the same when comparing the entire intervention group, pre vs. post (n = 31). The corrected effect sizes for split groups (e.g., low vs. high engagement or low vs. high SES) tended to be lower by 0.01 to 0.03. Putting aside spirituality (will revisit this topic) the hope effect sizes are comparable, or superior to those reported in a recent meta-analytic review of youth psychotherapy. Weisz et al. (2017) found a mean ES of 0.46 overall across all youth problems.

#### Depression

Weisz et al. reported a mean ES of 0.29 for treatments of youth depression. Our data show an ES of 0.38 for overall depression scores. Among highly engaged participants, the ES rose to 0.55 (Hedges g = 0.53). Weisz et al. noted that historically the “treatment of depression showed the most disappointing effects…the findings suggest that depression may be an appropriate priority for future treatment development and evaluation” (p. 93). Cognitive-Behavioral Therapy (CBT) is the most popular method for treating mild to moderate depression. More than 80% of published studies on the treatment of youth depression involve CBT (Hetrick et al., 2015). It can be argued that CBT most directly targets cognition and the mastery system, partially impacts survival (coping) processes, and has only minimal impact on attachment or spirituality (Bosmans, 2016; Pilgrim, 2011; Stewart-Sicking, 2015).

#### Anxiety

We did not anticipate a lack of change in anxiety levels. Interestingly, Survival Hope demonstrated the greatest magnitude of sub-scale change. Survival Hope, as defined in our model, includes self-regulation and liberation beliefs. The selected measure of anxiety (RCMAS; Reynolds and Richards, 2008) reflects a focus on autonomic arousal and social unease. It is possible that our hope intervention, while inclusive of a “survival/coping” dimension, fosters more activation than deactivation, by strengthening liberation beliefs (an expansion of limits). A secondary analysis of the Survival Hope subscales confirmed this hypothesis. We found a stronger effect for increased liberation beliefs as compared to self-regulation of fear and anxiety (*p* = 0.001, es = 0.66 vs. *p* = 0.024, es = 0.44, respectively).

#### Coping: support-seeking

The philosopher Marcel (1962) described openness as both the roots and fruits of fundamental hope. Lynch (1965) asserted that hope should not be seen as a “private resource” but a gift derived from “liberating relationships” and “collaborative mutuality.” The magnitude of change in social coping fell in between the hope and depression effects (moderate, es = 0.44). Group means for all five questions comprising the social coping variable trended upwards following the intervention. The greatest increase was for item 4, “reached out to a good problem solver.” Maladaptive social problem-solving strategies, including a limited generation of responses, characterize teens who engage in self-injurious behavior (Nock and Mendes, 2008), those with ADHD (Sibley et al., 2010) as well as adolescents with pre-natal exposure to alcohol (McGee et al., 2008). Thomassin et al. (2017) found that support-seeking mediated the link between emotion expression deficits and non-suicidal self-injury. Generally speaking, one of the aims of the hope intervention is to foster a more open, trusting, and socially engaged attitude. Social coping scores increased to a greater degree for lower SES participants. Some studies have shown that family economic and educational hardships can precipitate avoidant coping, higher levels of social distress and less effective pre-frontal control over negative emotions, particularly during simulated social exclusion experiments (Coan et al., 2015; Yanagisawa et al., 2013). However, there is also evidence that individuals from lower SES families often encounter more risk factors and accrue more benefits from protective factors (Wills et al., 1995).

#### Self-acceptance

We designed the groups to impact *being* as well as *doing*. Fundamental hope encompasses action tendencies and character. Eliott (2005, p. 38) observed that “hope does seem to be part of who deem ourselves to be.” Larsen and Stege (2012) discovered that “supportive identity development,” including fostering a sense of worthiness, was critical for instilling hope in counseling sessions. In this study, self-acceptance scores increased significantly for the treatment group. The change was most obvious for low SES participants. Presumably, self-acceptance is more challenging for individuals with limited resources (e.g., illness, handicap, poverty, minority status). Conversely, greater self-acceptance can be a source of hope in the presence of these or other risk factors. Among African American youth, Butler-Barnes et al. (2013) found that self-acceptance and self-efficacy were equally important in predicting academic persistence, but the former emerged as the strongest correlate of racial pride.

### Efficacy, effectiveness, and impact

What metrics should apply in evaluating the cost and benefits of a hope-based intervention? Demands for efficacy analysis (effect sizes) are becoming the norm for reporting intervention studies (Sapp, 2017). Effect sizes reflect the causal magnitude of an intervention at the point of contact, typically under highly controlled conditions (e.g., an RCT). Effectiveness is a different metric, defined as the “performance” of an intervention in real world situations. Singal et al. (2014) observe that any intervention study can be plotted somewhere along an efficacy—effectiveness continuum (from idealized to practical). The present study is closer to the ideal end of the spectrum. Abrams et al. (1996) define *impact* as a product of reach (percent penetration into a target population) and efficacy.

Combining these perspectives, a highly impactful hope intervention is possible using a group format (vs. individual counseling), delivered by paraprofessionals (vs. professional counselors), that demonstrates efficacy and effectiveness. The potential for use by paraprofessionals relates directly to the problem of reach or penetration. A recent Kaiser Family Foundation (2017) report showed a 56% nationwide gap between the need and availability of mental health providers. We can envision modifications that may increase both efficacy and effectiveness, including between session exercises, follow-up contact with hope mentors, or booster sessions. One method for addressing the practical challenges of extended long-term treatments may be to utilize brief virtual (telehealth) sessions, or an online resource center, with or without the addition of post-treatment exercises.

## Limitations

There are several limitations to this study. Our sample was predominately Caucasian and middle class. The intervention, while adequate from a statistical power perspective for a pilot study (1 - β = 0.76), was limited to 31 participants. In subsequent trials, a minimum of 35 participants in the treatment group should ensure a power level of 0.80. We excluded severely depressed, suicidal youth, and those taking psychiatric drugs or reporting a history of psychosis. For ethical reasons, we suggest the continued use of these exclusionary criteria when delivering this intervention in educational or other non-clinical settings. Moreover, in non-clinical settings that utilize lay providers (e.g., college or graduate students), it is imperative that adequate training and supervision is provided. Ultimately, it may be possible to institute a certification process for this intervention. Within clinical settings, we recommend that best practices are followed such as pre-trial disclosure of a need to inform parents and healthcare providers for individuals reporting high levels of depression or imminent suicidal risk. In general, more trials are needed with more heterogeneous samples, clinical and non-clinical.

We instituted an intensive training program, spanning nearly a year. We provided 3 h of supervision per module. Three hours of supervision per module appears reasonable under most circumstances (lay or professional providers). We are currently conducting a trial in Africa with a shorter training period of 30 h, employing a “train-the-trainers” model. Further research needs to be conducted, exploring different training lengths with more or less experienced providers. It is worth noting that less experienced providers may require a longer training period but will deliver the intervention at a reduced cost.

### Spirituality

We did not detect a significant change in Spiritual Presence. Perhaps teen participants interpreted our “spiritual” questions as referring to “God” or “religion” despite our best efforts to enlarge the discussion. It is possible that shifts in certain aspects of spirituality such as “presence” may be too difficult to measure with self-reports or require a more extended intervention (Hall et al., 2009). When we removed presence from our composite spiritual variable, and summed assurance (coping support) and inspiration (empowerment), the pre vs. post difference was significant.

## Concluding observations

A motivational and emotional account of hope highlights its role in addressing non-negotiable mastery, attachment, and survival needs on the edge of possibility. Hope lies between need and desire, present and future. Within this liminal space, three different forms of hope may emerge: fundamental (basic) hope, long-term aspirations, and event-activated state hopes. All three hopes share the qualities of animating as well as sustaining thoughts and actions. Averill et al.’s (1990) study of hope metaphors uncovered references to a gas or airy substance that provides a “lift”. The word [hope] in Hebrew (Tikvah) refers to a woven cord, i.e., something to be grasped in the process of mental-time travel. Hope has also been compared to an anchor; a resource for maintaining a fixed orientation in a liminal space threatened by potential disturbance.

Further work is needed to clarify the interactions among these different hopes. Whatever the findings, it is reasonable to presume that the character strength of fundamental hope impacts both long-term aspirations and the emergence (or non-emergence) of state hope in the context of potential harm, loss, or challenge. This is a compelling justification for an intervention targeting fundamental hope in youth. We might compare the hope intervention to a broad-spectrum immunization program, designed to protect the individual against despair arising from threats to the needs for mastery, attachment, survival, and spirituality, listed in this order to suggest a “critical m.a.s.s”.

## Data Availability

The raw data supporting the conclusions of this article will be made available by the authors, without undue reservation.

## References

[ref1] Abrams D. B. Orleans C. T. Niaura R. S. Goldstein M. G. Prochaska J. O. Velicer W. (1996). Integrating individual and public health perspectives for treatment of tobacco dependence under managed health care: a combined stepped-care and matching model. Ann. Behav. Med. 18, 290–304. doi: 10.1007/BF02895291, PMID: 18425675

[ref2] Abramson L. Y. Alloy L. B. Metalsky G. I. (1990). “The hopelessness theory of depression: current status and future directions” in Psychological and biological approaches to emotion. eds. Stein N. L. Leventhal B. Trabasso T. (Hillsdale, NJ, England: Lawrence Erlbaum Associates, Inc.), 5–21.

[ref3] Abramson L. Y. Seligman M. E. Teasdale J. D. (1978). Learned helplessness in humans: critique and reformulation. J. Abnorm. Psychol. 87, 49–74. doi: 10.1037/0021-843X.87.1.49, PMID: 649856

[ref4] Achenbach T. Rescorla L. (2000). Child behavior checklist. Burlington, VT: ASEBA.

[ref5] Amit B. H. Krivoy A. Mansbach-Kleinfeld I. Zalsman G. Ponizovsky A. M. Hoshen M.. (2014). Religiosity is a protective factor against self-injurious thoughts and behaviors in Jewish adolescents: findings from a nationally representative survey. Eur. Psychiatry 29, 509–513. doi: 10.1016/j.eurpsy.2014.04.005, PMID: 24908151

[ref6] Auerbach R. P. Millner A. J. Stewart J. G. Esposito E. C. (2015). Identifying differences between depressed adolescent suicide ideators and attempters. J. Affect. Disord. 186, 127–133. doi: 10.1016/j.jad.2015.06.031, PMID: 26233323 PMC4565772

[ref7] Averill J. R. Catlin G. Chon K. K. (1990). Rules of hope. New York: Springer-Verlag.

[ref9001] Barton Y. A. Miller L. Wickramaratne P. Gameroff M. J. Weissman M. M. (2013). Religious attendance and social adjustment as protective against depression: A 10-year prospective study. J. Affect. Disord. 146, 53–57. doi: 10.1016/j.jad.2012.08.037 22959684 PMC3582716

[ref9] Beck J. S. Bieling P. J. Grant V. V. (2012). “Cognitive therapy” in The art and science of brief psychotherapies: An illustrated guide. eds. Dewan M. J. Steenbarger B. N. Greenberg R. P. Dewan M. J. Steenbarger B. N. Greenberg R. P. (Arlington, VA, US: American Psychiatric Publishing, Inc.), 45–81.

[ref10] Bion W. R. (2004). Experience in groups. New York: Taylor and Francis.

[ref11] Bosmans G. (2016). Cognitive behavior therapy for children and adolescents: can attachment theory contribute to its efficacy? Clin. Child. Fam. Psychol. Rev. 19, 310–328. doi: 10.1007/s10567-016-0212-3, PMID: 27576548

[ref9002] Bowlby J. (1969). Attachment and Loss, *Vol. 1*. Basic Books.

[ref12] Breznitz S. (1986). “The effect of hope on coping with stress” in Dynamics of stress: Physiological, psychological, and social perspectives. eds. Appley M. H. Trumbull R. Appley M. H. Trumbull R. (New York, NY, US: Plenum Press), 295–306.

[ref14] Buchanan C. L. (2008). Making Hope happen for students receiving special education services [ProQuest Information & Learning]. In Dissertation abstracts international: Section B: The sciences and engineering (Vol. 69, Issue 3–B, p. 1943).

[ref15] Burson A. Crocker J. Mischkowski D. (2012). Two types of value-affirmation: implications for self-control following social exclusion. Soc. Psychol. Personal. Sci. 3, 510–516. doi: 10.1177/1948550611427773

[ref16] Butler-Barnes S. T. Chavous T. M. Hurd N. Varner F. (2013). African American adolescents’ academic persistence: a strengths-based approach. J. Youth Adolesc. 42, 1443–1458. doi: 10.1007/s10964-013-9962-0, PMID: 23700259

[ref17] Cairns K. E. Yap M. H. Pilkington P. D. Jorm A. F. (2014). Risk and protective factors for depression that adolescents can modify: a systematic review and meta-analysis of longitudinal studies. J. Affect. Disord. 169, 61–75. doi: 10.1016/j.jad.2014.08.006, PMID: 25154536

[ref18] Campbell J. (1991). The power of myth. New York: Anchor books.

[ref19] Caprara G. V. Gerbino M. Paciello M. Di Giunta L. Pastorelli C. (2010). Counteracting depression and delinquency in late adolescence: the role of regulatory emotional and interpersonal self-efficacy beliefs. Eur. Psychol. 15, 34–48. doi: 10.1027/1016-9040/a000004

[ref20] Carter H. (1953). Where main street meets the river. New York: Rinehart.

[ref9003] Centers for Disease Control and Prevention (2016). Youth Risk Behavior Survey. Available at: https://www.cdc.gov/yrbs/files/2015/pdf/2015_hs_questionnaire.pdf

[ref21] Coan J. A. Gonzalez M. Z. Chango J. Allen J. P. Beckes L. (2015). Adolescent neighborhood quality predicts adult dACC response to social exclusion. Soc. Cogn. Affect. Neurosci. 10, 921–928. doi: 10.1093/scan/nsu137, PMID: 25349459 PMC4483560

[ref22] Compas B. E. Connor-Smith J. Jaser S. S. (2004). Temperament, stress reactivity, and coping: implications for depression in childhood and adolescence. J. Clin. Child Adolesc. Psychol. 33, 21–31. doi: 10.1207/S15374424JCCP3301_3, PMID: 15028538

[ref24] Daniel S. S. Goldston D. B. Erkanli A. Heilbron N. Franklin J. C. (2017). Prospective study of major loss life events and risk for suicidal thoughts and behaviors among adolescents and young adults. Suicide Life Threat. Behav. 47, 436–449. doi: 10.1111/sltb.12305 27862201 PMC6485934

[ref26] Dew R. E. Daniel S. S. Goldston D. B. McCall W. V. Kuchibhatla M. Schleifer C.. (2010). A prospective study of religion/spirituality and depressive symptoms among adolescent psychiatric patients. J. Affect. Disord. 120, 149–157. doi: 10.1016/j.jad.2009.04.029, PMID: 19450882

[ref27] Dubois N. Beauvois J. (2005). Normativeness and individualism. Eur. J. Soc. Psychol. 35, 123–146. doi: 10.1002/ejsp.236

[ref9004] Eliott J. A. (2005). What have we done with hope? A brief history. In Interdisciplinary perspectives on hope. Nova Science Publishers. 3–45.

[ref28] Emmons R. A. (1999). The psychology of ultimate concerns. New York: Guilford.

[ref29] Erikson E. H. (1950). Childhood and society. New York: Norton.

[ref30] Ferrer R. A. Shmueli D. Bergman H. E. Harris P. R. Klein W. P. (2012). Effects of self-affirmation on implementation intentions and the moderating role of affect. Soc. Psychol. Personal. Sci. 3, 300–307. doi: 10.1177/1948550611419265

[ref31] Folkman S. (2010). Stress, coping, and hope. Psycho-Oncology 19, 901–908. doi: 10.1002/pon.1836, PMID: 20799373

[ref32] Folkman S. Lazarus R. S. (1988). Ways of coping questionnaire. Palo Alto, CA: Consulting Psychological Press.

[ref33] Fowler J. W. (1981). Stages of faith. New York: Harper and Row.

[ref34] Franklin J. C. Ribeiro J. D. Fox K. R. Bentley K. H. Kleiman E. M. Huang X.. (2017). Risk factors for suicidal thoughts and behaviors: a meta-analysis of 50 years of research. Psychol. Bull. 143, 187–232. doi: 10.1037/bul0000084, PMID: 27841450

[ref36] Gallagher M. W. Long L. J. Richardson A. D’Souza J. Boswell J. F. Farchione T. J.. (2020). Examining hope as a transdiagnostic mechanism of change across anxiety disorders and CBT treatment protocols. Behav. Ther. 51, 190–202. doi: 10.1016/j.beth.2019.06.001, PMID: 32005336 PMC7000132

[ref38] Hall T. W. Fujikawa A. Halcrow S. R. Hill P. C. Delane H. (2009). Attachment to god and implicit spirituality: clarifying correspondence and compensation models. J. Psychol. Theol. 37, 227–244. doi: 10.1177/009164710903700401

[ref39] Harris P. R. (2011). Self-affirmation and the self-regulation of health behavior change. Self Identity 10, 304–314. doi: 10.1080/15298868.2010.517963

[ref40] Harvard University (2018) Harvard ED portal Mentor program. Available at: https://edportal.harvard.edu/mentor (accessed April 9, 2018)

[ref41] Hawton K. Comabella C. Haw C. Saunders K. (2013). Risk factors for suicide in individuals with depression: a systematic review. J. Affect. Disord. 147, 17–28. doi: 10.1016/j.jad.2013.01.004, PMID: 23411024

[ref42] Hedges L. V. Olkin I. (1985). Statistical methods for meta-analysis. New York, NY: Academic Press.

[ref9005] Hetrick S. E. Cox G. R. Fisher C. A. Bhar S. S. Rice S. M. Davey C. G.. (2015). Back to basics: Could behavioral therapy be a good treatment option for youth depression?. A critical review. Early Interv. Psychiatry. 9, 93–99. doi: 10.1111/eip.12142 24698212

[ref44] Ho T. C. Gifuni A. J. Gotlib I. H. (2022). Psychobiological risk factors for suicidal thoughts and behaviors in adolescence: a consideration of the role of puberty. Mol. Psychiatry 27, 606–623. doi: 10.1038/s41380-021-01171-5, PMID: 34117365 PMC8960417

[ref45] Hoberman H. M. (1989). Completed suicide in children and adolescents: a review. Resid. Treat. Child. Youth 7, 61–88. doi: 10.1300/J007v07n01_04

[ref46] Holder M. K. Blaustein J. D. (2014). Puberty and adolescence as a time of vulnerability to stressors that alter neurobehavioral processes. Front. Neuroendocrinol. 35, 89–110. doi: 10.1016/j.yfrne.2013.10.004, PMID: 24184692 PMC3946873

[ref47] Horton S. E. Hughes J. L. King J. D. Kennard B. D. Westers N. J. Mayes T. L.. (2016). Preliminary examination of the interpersonal psychological theory of suicide in an adolescent clinical sample. J. Abnorm. Child Psychol. 44, 1133–1144. doi: 10.1007/s10802-015-0109-5, PMID: 26667025

[ref48] James K. H. (2017). The importance of handwriting experience on the development of the literate brain. Curr. Dir. Psychol. Sci. 26, 502–508. doi: 10.1177/0963721417709821

[ref49] Jayne K. M. Ray D. C. (2016). Child-centered play therapy as a comprehensive school counseling approach: directions for research and practice. Pers.-Centered Exp. Psychother. 15, 5–18. doi: 10.1080/14779757.2015.1132757

[ref50] Jiang X. Otis K. L. Weber M. Huebner E. S. (2018). “Hope and adolescent mental health” in The Oxford handbook of hope. eds. Gallagher M. W. Lopez S. J. (Oxford: Oxford University Press), 299–312.

[ref51] Joiner T. E. (2002). “Depression in its interpersonal context” in Handbook of depression. eds. Gotlib I. H. Hammen C. L. (New York: Guilford), 295–313.

[ref52] Jones A. C. Schinka K. C. van Dulmen M. M. Bossarte R. M. Swahn M. H. (2011). Changes in loneliness during middle childhood predict risk for adolescent suicidality indirectly through mental health problems. J. Clin. Child Adolesc. Psychol. 40, 818–824. doi: 10.1080/15374416.2011.614585, PMID: 22023273

[ref53] Kaiser Family Foundation (2017) Statistics. Available at: https://www.kff.org/other/state-indicator/mental-health-care-health-professional-shortage-areas (accessed January 11, 2018)

[ref54] Kaye S. Thomson P. (2006). Philosophy for teens. Waco, TX: Prufrock Press.

[ref55] Kazdin A. E. Rodgers A. Colbus D. (1986). The hopelessness scale for children: psychometric characteristics and concurrent validity. J. Consult. Clin. Psychol. 54, 241–245. doi: 10.1037/0022-006X.54.2.241, PMID: 3700812

[ref56] Kim S. Esquivel G. B. (2011). Adolescent spirituality and resilience: theory, research, and educational practices. Psychol. Sch. 48, 755–765. doi: 10.1002/pits.20582

[ref58] Klein J. B. Jacobs R. H. Reinecke M. A. (2007). Cognitive-behavioral therapy for adolescent depression: a Meta-analytic investigation of changes in effect-size estimates. J. Am. Acad. Child Adolesc. Psychiatry 46, 1403–1413. doi: 10.1097/chi.0b013e3180592aaa, PMID: 18049290 PMC2270481

[ref59] Klonsky E. D. May A. M. (2015). The three-step theory (3ST): a new theory of suicide rooted in the 'ideation-to-action' framework. Int. J. Cogn. Ther. 8, 114–129. doi: 10.1521/ijct.2015.8.2.114

[ref60] Kohut H. (1971). The analysis of the self. New York: International Universities Press.

[ref61] Kouros C. D. Garber J. (2014). Trajectories of individual depressive symptoms in adolescents: gender and family relationships as predictors. Dev. Psychol. 50, 2633–2643. doi: 10.1037/a0038190, PMID: 25329553 PMC4591045

[ref62] Kovacs M. (1985). The Children’s depression inventory (CDI). Psychopharmacol. Bull. 21, 995–998, PMID: 4089116

[ref63] Kratochvil C. J. Simons A. Vitiello B. Walkup J. Emslie G. Rosenberg D.. (2006). A multisite psychotherapy and medication trial for depressed adolescents: background and benefits. Cogn. Behav. Pract. 12, 159–165. doi: 10.1016/S1077-7229(05)80021-5

[ref64] Larsen D. J. Stege R. (2012). Client accounts of hope in early counseling sessions: a qualitative study. J. Couns. Dev. 90, 45–54. doi: 10.1111/j.1556-6676.2012.00007.x

[ref65] Li D. Li X. Wang Y. Bao Z. (2016). Parenting and Chinese adolescent suicidal ideation and suicide attempts: the mediating role of hopelessness. J. Child Fam. Stud. 25, 1397–1407. doi: 10.1007/s10826-015-0334-0

[ref66] Lowe P. A. (2015). The revised Children’s manifest anxiety scale–-second edition short form: examination of the psychometric properties of a brief measure of general anxiety in a sample of children and adolescents. J. Psychoeduc. Assess. 33, 719–730. doi: 10.1177/0734282915580763

[ref67] Lynch W. F. (1965). Images of hope: Imagination as healer of the hopeless. Baltimore, MD: Helicon Press.

[ref68] Marcel G. (1962). Homo Viator: Introduction to a metaphysics of hope. (Cauford E., Trans.). New York: Harper and Row (Original work published 1944).

[ref69] Marques S. C. Lopez S. J. Pais-Ribeiro J. L. (2011). “Building hope for the future”: a program to foster strengths in middle-school students. J. Happiness Stud. 12, 139–152. doi: 10.1007/s10902-009-9180-3

[ref71] May R. (1975). The courage to create. New York: Norton.

[ref72] McAdams D. P. McLean K. C. (2013). Narrative identity. Curr. Dir. Psychol. Sci. 22, 233–238. doi: 10.1177/0963721413475622

[ref73] McCluskey A. T. Smith E. M. (2002). Mary McLeod Bethune: Building a better world, selected essays and documents. Bloomington, IN: University of Indiana.

[ref74] McElhaney K. B. Antonishak J. Allen J. P. (2008). They like me, they like me not': popularity and adolescents' perceptions of acceptance predicting social functioning over time. Child Dev. 79, 720–731. doi: 10.1111/j.1467-8624.2008.01153.x, PMID: 18489423 PMC3073367

[ref75] McGee C. L. Fryer S. L. Bjorkquist O. A. Mattson S. N. Riley E. P. (2008). Deficits in social problem solving in adolescents with prenatal exposure to alcohol. Am. J. Drug Alcohol Abuse 34, 423–431. doi: 10.1080/00952990802122630, PMID: 18584572

[ref76] Menninger K. (1959). The academic lecture: Hope. Am. J. Psychiatry 116, 481–491. doi: 10.1176/ajp.116.6.481 13104684

[ref77] Miller L. Barton Y. A. (2015). Developmental depression in adolescents: a potential sub- type based on neural correlates and comorbidity. J. Relig. Health 54, 817–828. doi: 10.1007/s10943-015-0047-0, PMID: 25877664

[ref78] Moltmann J. (1993). Theology of hope. Minneapolis, MN: Fortress Press.

[ref79] Mowrer O. H. (1960). Learning theory and behavior. New York: Wiley and Sons.

[ref80] Mueller P. A. Oppenheimer D. M. (2014). The pen is mightier than the keyboard: advantages of longhand over laptop note taking. Psychol. Sci. 25, 1159–1168. doi: 10.1177/0956797614524581 24760141

[ref9006] Ng M. Y. DiVasto K. A. Cootner S. Gonzalez N. Weisz J. R. (2020). What do 30 years of randomized trials tell us about how psychotherapy improves youth depression? A systematic review of candidate mediators. Clin. Psychol.: Sci. Pract. doi: 10.1111/cpsp.12367

[ref82] NIH (2015). Suicide statistics for 10-to 14-year-olds. Available at: https://www.nimh.nih.gov/health/statistics/suicide.shtml#part_154969 (accessed January 10, 2015)

[ref83] NIMH (2014a). Prevalence rates of major depressive disorder among adolescents. Available at: http://www.nimh.nih.gov/health/statistics/prevalence/major-depression-among-adolescents.shtml (accessed December 5, 2014)

[ref84] NIMH (2014b). Prevalence rates of major depressive disorder among children. Available at: http://www.nimh.nih.gov/health/statistics/prevalence/any-mood-disorder-in-children.shtml (accessed December 5, 2014)

[ref85] Nock M. K. Mendes W. B. (2008). Physiological arousal, distress tolerance, and social problem-solving deficits among adolescent self-injurers. J. Consult. Clin. Psychol. 76, 28–38. doi: 10.1037/0022-006X.76.1.28, PMID: 18229980

[ref9007] Norcross J. C. Goldfried M. R. (2005). Handbook of psychotherapy integration. 2nd ed. Oxford University Press.

[ref86] Patterson J. M. McCubbin H. I. (1987). Adolescent coping style and behaviors: conceptualization and measurement. J. Adolesc. 10, 163–186. doi: 10.1016/S0140-1971(87)80086-6, PMID: 3611466

[ref87] Petito F. Cummins R. A. (2000). Quality of life in adolescence: the role of perceived control, parenting style, and social support. Behav. Chang. 17, 196–207. doi: 10.1375/bech.17.3.196

[ref88] Piers E. V. Herzberg D. S. (2002). Piers-Harris Children’s self-concept scale-second edition manual. Los Angeles, CA: Western Psychological Services.

[ref89] Pilgrim D. (2011). The hegemony of cognitive-behavior therapy in modern mental health care. Health Sociol. Rev. 20, 120–132. doi: 10.5172/hesr.2011.20.2.120

[ref90] Pruyser W. (1986). Maintaining hope in adversity. Pastor. Psychol. 35, 120–131. doi: 10.1007/BF01768711

[ref91] Quiroga C. V. Janosz M. Bisset S. Morin A. S. (2013). Early adolescent depression symptoms and school dropout: mediating processes involving self-reported academic competence and achievement. J. Educ. Psychol. 105, 552–560. doi: 10.1037/a0031524

[ref92] Reynolds N. Mrug S. Hensler M. Guion K. Madan-Swain A. (2014). Spiritual coping and adjustment in adolescents with chronic illness: a 2-year prospective study. J. Pediatr. Psychol. 39, 542–551. doi: 10.1093/jpepsy/jsu011, PMID: 24648256

[ref93] Reynolds B. O. Richards C. R. (2008). RCMAS-second edition manual. Los Angeles, CA: Western Psychological Services.

[ref9008] Rogers C. R. (1953). The interest in the practice of psychotherapy. Am. Psychol. 8, 48–50. doi: 10.1037/h0062136

[ref9009] Rogers C. R. (1985). Towards a More Human Science of the Person. J. Humanist. Psychol. 25:7. doi: 10.1177/0022167885254002

[ref94] Rokeach M. (1973). The nature of human values. New York: The Free Press.

[ref96] Rychlak J. F. (1993). Behavior as telosponsivity rather than responsivity. J. Mind Behav. 14, 365–372.

[ref97] Ryff C. D. Keyes C. M. (1995). The structure of psychological well-being revisited. J. Pers. Soc. Psychol. 69, 719–727. doi: 10.1037/0022-3514.69.4.719, PMID: 7473027

[ref98] Sapp M. (2017). Primer on effect sizes, simple research designs, and confidence intervals [e-book]. Springfield, IL, US: Charles C Thomas Publisher.

[ref99] Schmutte P. S. Ryff C. D. (1997). Personality and well-being: reexamining methods and meanings. J. Pers. Soc. Psychol. 73, 549–559. doi: 10.1037/0022-3514.73.3.549, PMID: 9294901

[ref101] Scioli A. Biller H. B. (2009). Hope in the age of anxiety. New York: Oxford University Press.

[ref102] Scioli A. MacPherson N. Murphy R. Adachi-Mejia A. Lyons K. (2016) Factor analysis and structural modeling of hope scores in children, adolescents, and emerging adults. Poster session presented at the annual meeting of APA division 36, New York.

[ref103] Scioli A. Ricci M. Nyugen T. Scioli E. R. (2011). Hope: its nature and measurement. Psychol. Relig. Spiritual. 3, 78–97. doi: 10.1037/a0020903

[ref9010] Seligman M. E. Railton P. Baumeister R. F. Sripada C. (2016). Homo prospectus. Oxford University Press.

[ref104] Sherman D. K. (2013). Self-affirmation: understanding the effects. Soc. Personal. Psychol. Compass 7, 834–845. doi: 10.1111/spc3.12072

[ref105] Shumaker D. (2012). An existential–integrative treatment of anxious and depressed adolescents. J. Humanist. Psychol. 52, 375–400. doi: 10.1177/0022167811422947

[ref106] Sibley M. H. Evans S. W. Serpell Z. N. (2010). Social cognition and interpersonal impairment in young adolescents with ADHD. J. Psychopathol. Behav. Assess. 32, 193–202. doi: 10.1007/s10862-009-9152-2

[ref107] Singal A. G. Higgins P. R. Waljee A. K. (2014). A primer on effectiveness and efficacy trials. Clin. Transl. Gastroenterol. 5:e45. doi: 10.1038/ctg.2013.13, PMID: 24384867 PMC3912314

[ref108] Smith H. (2006). Why religion matters. New York: Harper-Collins.

[ref109] Snyder C. R. Harris C. Anderson J. R. Holleran S. A. Irving L. M. Sigmon S. T.. (1991). The will and ways: development and validation of an individual differences measure of hope. J. Pers. Soc. Psychol. 60, 570–585. doi: 10.1037/0022-3514.60.4.570, PMID: 2037968

[ref110] Stewart-Sicking J. (2015). Cognitive therapy and the punctual self: using an ascetical framework to critique approaches to psychotherapy. Pastor. Psychol. 64, 111–122. doi: 10.1007/s11089-013-0588-7

[ref111] Stoddard S. A. Henly S. J. Sieving R. E. Bolland J. (2011). Social connections, trajectories of hopelessness, and serious violence in impoverished urban youth. J. Youth Adolesc. 40, 278–295. doi: 10.1007/s10964-010-9580-z, PMID: 20690037 PMC3105375

[ref112] Stotland E. (1969). The psychology of hope. San Francisco, CA: Jossey-Bass.

[ref113] Talib M. A. Abdollahi A. (2017). Spirituality moderates hopelessness, depression, and suicidal behavior among Malaysian adolescents. J. Relig. Health 56, 784–795. doi: 10.1007/s10943-015-0133-3, PMID: 26429729

[ref114] Thomassin K. Guérin Marion C. Venasse M. Shaffer A. (2017). Specific coping strategies moderate the link between emotion expression deficits and non-suicidal self-injury in an inpatient sample of adolescents. Child Adolesc. Psychiatry Ment. Health 11:21. doi: 10.1186/s13034-017-0158-3, PMID: 28413442 PMC5390354

[ref115] Thurman S. K. Torsney B. M. (2014). “Meditation, mindfulness and executive functions in children and adolescents” in Psychology of meditation. eds. Singh N. N. Singh N. N. (Hauppauge, NY, US: Nova Science Publishers), 187–207.

[ref116] Tillich P. (1952). The courage to be. New Haven, CT: Yale University Press.

[ref117] University of Notre Dame (2018) Notre Dame Vision Academy. Available at: https://icl.nd.edu/notre-dame-vision/learn-more/for-high-school-youth/ (accessed April 9, 2018)

[ref118] University of the Redlands (2016) College and high school partnership. Available at: http://ochtamalemagazine.net/the-college-high-school-alliance-mentoring-program-and-service-champs/ (accessed April 9, 2018)

[ref119] Venta A. Hatkevich C. Sharp C. Rotenberg K. (2017). Low emotional trust in mothers is associated with increased suicide attempts in inpatient adolescents with depressive symptoms. J. Soc. Clin. Psychol. 36, 221–237. doi: 10.1521/jscp.2017.36.3.221

[ref120] Vygotsky L. (1978). Mind in society. Cambridge, MA: Harvard University Press.

[ref121] Webb C. A. Auerbach R. P. DeRubeis R. J. (2012). Processes of change in CBT of adolescent depression: review and recommendations. J. Clin. Child Adolesc. Psychol. 41, 654–665. doi: 10.1080/15374416.2012.704842, PMID: 22867130

[ref122] Wei J. Carroll R. J. Harden K. K. Wu G. (2012). Comparisons of treatment means when factors do not interact in two-factorial studies. Amino Acids (Vienna) 42, 2031–2035. doi: 10.1007/s00726-011-0924-0, PMID: 21547361 PMC3199378

[ref9011] Weisz J. R. Kuppens S. Ng M. Y. Eckshtain D. Ugueto A. M. Vaughn-Coaxum R.. (2017). What five decades of research tells us about the effects of youth psychological therapy: A multilevel meta-analysis and implications for science and practice. Am. Psychol. 72, 79–117. doi: 10.1037/a0040360 28221063

[ref123] Weisz J. R. McCarty C. A. Valeri S. M. (2006). Effects of psychotherapy for depression in children and adolescents: a meta-analysis. Psychol. Bull. 132, 132–149. doi: 10.1037/0033-2909.132.1.132, PMID: 16435960 PMC2150594

[ref124] Weisz J. R. Southam-Gerow M. A. McCarty C. A. (2001). Control-related beliefs and depressive symptoms in clinic-referred children and adolescents: developmental differences and model specificity. J. Abnorm. Psychol. 110, 97–109. doi: 10.1037/0021-843X.110.1.97 11261405

[ref125] Wiitala W. L. Dansereau D. F. (2004). Using popular quotations to enhance therapeutic writing. J. Coll. Couns. 7, 187–191. doi: 10.1002/j.2161-1882.2004.tb00250.x

[ref126] Wilkinson P. Dubicka B. Kelvin R. Roberts C. Goodyer I. (2009). Treated depression in adolescents: predictors of outcome at 28 weeks. Br. J. Psychiatry 194, 334–341. doi: 10.1192/bjp.bp.108.052381, PMID: 19336785

[ref127] Wills T. A. McNamara G. Vaccaro D. (1995). Parental education related to adolescent stress-coping and substance use: development of a mediational model. Health Psychol. 14, 464–478. doi: 10.1037/0278-6133.14.5.464, PMID: 7498118

[ref128] Wittgenstein L. (1953/2009). Philosophical investigations. West Sussex, UK: Wiley–Blackwell.

[ref129] Wright B. A. Shontz F. (1968). Process and tasks in hoping. Rehabil. Lit. 29, 322–331, PMID: 4235139

[ref130] Yanagisawa K. Masui K. Furutani K. Nomura M. Yoshida H. Ura M. (2013). Family socioeconomic status modulates the coping-related neural response of offspring. Soc. Cogn. Affect. Neurosci. 8, 617–622. doi: 10.1093/scan/nss039, PMID: 22446300 PMC3739906

