# Supplementary material for: A hope intervention for adolescents: a randomized controlled trial delivered by paraprofessionals
Source: Front Psychol. 2025 May 5;16:1528504. doi: 10.3389/fpsyg.2025.1528504 (PMC12087476; doi:10.3389/fpsyg.2025.1528504)
Supplement: Supplementary file 1 [file Data_Sheet_1.docx]

**Supplementary File** (further information regarding the hope intervention described in Scioli et al…A hope intervention for adolescents…)

**Title and Authors**

**A Hope Intervention for Adolescents: A Randomized Controlled Trial Delivered by Paraprofessionals**

Anthony Scioli, Nathan MacPherson, Rachel Murphy, Tyler Gooding, and Micah Love

Keene State College – University System of New Hampshire

Kathleen D. Lyons and Anna M. Adachi–Mejia

Geisel School of Medicine at Dartmouth at the time of the study

Contact for Further Information

Anthony Scioli, Ph.D.

Professor of Clinical Psychology

Keene State College – University System of New Hampshire

Keene, New Hampshire – USA

Adjunct Clinical Graduate Faculty – Psychology Department

University of Rhode Island

Kingston, Rhode Island – USA

Email: [tscioli@keene.edu](mailto:tscioli@keene.edu)

See also The Hope Institute: www.gainhope.com

Workshop Copyright © 2012 Anthony Scioli

**Manual Summary: Scioli Hope Intervention**

**Introduction:** This manual describes a method for instilling fundamental hope in adolescents through a ten-session workshop. The individual sessions (modules) should take approximately 50 to 60 minutes. Various scheduling options are possible. However, my team and I prefer a five-week delivery schedule (5 weeks of 2 sessions per week).

**Broad Spectrum Approach**: Many psychotherapeutic approaches for children and teens focus on specific disorders such as Attention Deficit, Conduct Disorder, Anxiety, or Depression. *In contrast, this manual offers a broad-based program for building the character strength of “fundamental hope”.* Consider the analogy of broad-spectrum immunization. One treatment will address multiple diseases. In a similar fashion, this workshop on hope will build resistance to anxiety, depression, and psychological trauma. Fundamental hope is more than a palliative. It is a resource for actively engaging with the world and for coping with the vicissitudes of life. From this perspective, the cultivation of hope is comparable to the induction of a potent psycho-social-spiritual immune booster.

**Rationale for Hope**: Hope in its fullest sense encompasses the four greatest needs of a human being. These include attachment (trust, openness, and connection), survival (self-regulation and feelings of liberation), mastery (empowerment, ambition, and ideals), and spirituality (faith and higher support for attachment, survival and mastery). When we direct our attention to the youth of the world, it is not surprising that what we hope for them parallels these dimensions of hope. We want children to experience love, to do their best, to cope with adversity, and to live a meaningful life. In short, there is much wisdom in the old adage that hope is humanity’s “best medicine”.

**Workshop Sequence:** I sequenced the workshop topics to reflect the natural growth of hope. Attachments are the first seeds of hope. After attachment is established, survival skills are developed. Once these two pillars are in place, mastery is required. After mastery, there is a need for spiritual hope.

**Evidence-Based Programming:** Human behavior is complex. Aristotle noted that not everything that counts can be counted; not everything that can be counted is worth counting. Nevertheless, there is something to be said for accountability and striving for an “evidence – based” intervention. I include a hope pre-test and a hope post-test whenever we conduct a trial. I gather and analyze the data from all my workshops. This information helps my team and I to validate the work of “hope providers” while providing some hard data to support its effectiveness and continued use. Moreover, I use the data and feedback to make improvements in future versions of this manual.

**Overview of Content**

**Integrative Approach:** This workshop represents a multidisciplinary approach to hope. I combine psychological science with philosophy, spiritual writings, literature, history, and the arts. On the psychological side, I incorporate cognitive, behavioral, humanistic and psychoanalytic dimensions of hope, along with recent advances in meditation and hypnosis. The first two sessions feature aspects of attachment theory, one of the most influential scientific paradigms of the past two decades. This includes the work of John Bowlby, Mary Ainsworth, and Alan Schore on interpersonal and neuropsychological effects of anxious and insecure attachments as well as the writings of Erik Erikson (hope and basic trust), and psychoanalytic psychiatrist Heinz Kohut (twinship, mirroring, and idealizing needs). Other influences include psychologist Thomas Joiner’s interpersonal understanding of suicide, and the philosopher Gabriel Marcel’s writings on trust, faith, and hope. Some of the meditations were adapted from scripts developed by a leading hypnotherapist, Daniel Brown. These scholarly contributions appear in conjunction with passages from Aristotle, Henry Ward Beecher, the Bible, Torah and Koran as well as Buddhist and Hindu sacred texts, and African and Native America wisdom.

The sections on survival deal with self–regulation and liberation beliefs, two aspects of survival–based (or coping-focused) hope. These aspects reflect current research on mindfulness and mentalizing. The former refers to an attitude of acceptance that heightens awareness and fosters better self–regulation while the latter relates to meta–cognitive processes that draw on life history (memories and images) and context to provide perspective and flexibility (liberation). These survival skills follow quotes from Louise May Alcott, Tertullian, an African Proverb (Ghana), Jawaharlal Nehru as well as from Tolkien’s Twin Towers (the Lord of the Rings trilogy). An SOS exercise combines psychologist Rollo May’s ideas on the four kinds of destiny with research on ways of coping by psychologists Susan Folkman and Richard Lazarus, and the process of “reality surveillance” reported by parents of children dealing with a chronic illness.

The mastery lessons begin with historical examples of achievement that required a strong sense of purpose as well as help from others. There are reflections from Mary McLeod Bethune, Nelson Mandela, Roger Bannister and the Seneca Indians. The integrative life-skills exercise links hopeful mastery to a clear sense of values and includes a self–monitoring component for gauging value–consistent and value–inconsistent behaviors. The mastery meditation includes insights from psychologist Robert Emmons’ research on the sanctification of goals, the philosopher Gabriel Marcel’s writings on ultimate hope, and Heinz Kohut’s ideas on empowering the self through transformation of incorporated others.

The spirituality session addresses a need for faith in one more “centers of value”. This is a term borrowed from James Fowler’s influential writings on faith development. Another theme introduced in this session is the notion of engaging in a “hopeful task” or life mission. This concept draws from a number of sources, including the anthropologist Ernest Becker, Gandhi, and Martin Luther King. The reflections in this section expand the concept of spirituality to embrace family and human life (Albert Schweitzer), science (Carl Sagan), nature (Anne Frank) and art (Georgia O’Keefe). I also include quotes from Joseph Campbell, Gandhi, and the Dakota Indians. The integrative exercise will help participants identify their particular spiritual type (mystic, collaborator, follower, sufferer, etc.) and point them towards activities and careers consistent with these spiritual leanings.

Participants also receive a take-home handout (not covered in the workshop) of readings and music for the left and right brain, repeated across seven religious traditions. Through this diversity offering, participants can deepen an existing faith or enlarge their spiritual center.

**Whole Brain Perspective:** We repeat 4 kinds of activities in each of the modules: philosophical reflections, integrative exercises, meditative–hypnotic practices, and song writing. This combination is designed to address the whole brain. The *left-brain* is logical, detail–oriented, and verbal. The integrative exercises are primarily left–brain activities. The *right brain* is holistic in orientation, responds to symbols, images, and metaphors as well as meditative–hypnotic practices. The right brain responds to philosophy, religion, and spirituality. The philosophical reflections stimulate the left-brain (written material) and the right brain (metaphors and images). The meditative–hypnotic practices are right brain mediated.

Philosophical reflections provide guiding or organizing life principles, the integrative exercises impart skills, and the meditative-hypnotic practices help to consolidate and deepen each of the four building blocks of hope.

|  | ***Philosophical Reflections*** | ***Integrative Exercises*** | ***Meditative-Hypnotic Exercise*** |
| --- | --- | --- | --- |
| ***Mechanism of Change*** | **Guiding Life Principles** | **Skill Development** | **Consolidation & Deepening** |
| ***Level of Impact*** | **Mind Sets** | **Skill Sets** | **Mind-Body Connectivity** |
| ***Facet of Hope*** | **Hope as Seeing** | **Hope as Doing** | **Hope as Being** |

**Recommended for Workshop Leaders:** My Oxford book on hope is a comprehensive resource for any potential hope provider. It includes the theoretical, historical, scientific and literary foundations of this workshop. I highly recommend it as a companion text to this manual.

Scioli, A. & Biller, H. B. (2009). *Hope in the Age of Anxiety*. New York: Oxford University Press.

**Details of Session Contents**

**WEEK 1: ATTACHMENT I**

**Day 1 (60 minutes)**

**Philosophical Reflections:** Aristotle **●** Jewish Proverb **●** English Proverb **●** African Proverb

**●** Shakespeare

**Integrative Life-Skills:** What is Trust? ● Trust Detectors

**Day 2 (60 Minutes)**

**Integrative Life-Skills:** Hope Chests

**Meditative–Hypnotic Exercise:** Waves of Relaxation ● Meditation on Attachment

**WEEK 2: ATTACHMENT 2**

**Day 1 (60 Minutes)**

**Philosophical Reflections:** Ecclesiastes **●** Schweitzer **●** Kinyarwanda Proverb ● Beecher

● Akita

**Integrative Life-Skills:** Care Recruitment

**Day 2 (60 Minutes)**

**Integrative Life-Skills:** Mirrors and Role Models

**Meditative–Hypnotic Exercise:** Waves of Relaxation ● Meditation on Attached Survival

and Mastery

**WEEK 3: SURVIVAL**

**Day 1 (60 Minutes)**

**Philosophical Reflections:** Alcott **●** Tertullian ● African Proverb **●**Nehru **●**Tolkien

**Integrative Life-Skills:** Options for Coping

**Day 2 (60 Minutes)**

**Integrative Life-Skills:** Stretch your Potential

**Meditative–Hypnotic Exercise:** Waves of Relaxation ● Meditation on Survival

**WEEK 4 MASTERY**

**Day 1 (60 Minutes)**

**Philosophical Reflections:** Mary McLeod Bethune ● Mandela **●** Seneca Tribe **●**

Roger Bannister

**Integrative Life-Skills:** Steps to Success, Steps 1-3

**Day 2 (60 Minutes)**

**Integrative Life-Skills:** Steps to Success, Steps 4-5

**Meditative–Hypnotic Exercise:** Waves of Relaxation ● Meditation on Mastery

**WEEK 5 SPIRITUALITY**

**Day 1 (60 Minutes)**

**Philosophical Reflections:** Einstein ● Schweitzer ● Sagan ●Frank ●Plato ● O’Keefe

● Campbell ● Gandhi ● Dakota Tribe

**Integrative Life-Skills:** Assessment of Six Spiritual Types

**Day 2 (60 Minutes)**

**Integrative Life-Skills:** Activities and Careers for Spiritual Types ● Diversity Take-Home

Handout

**Meditative–Hypnotic Exercise:** Waves of Relaxation ● Meditation on Spirituality

**Further Orientation for Group Leaders**

**A. What is Hope?**

More than ever the world needs hope. These are dark times. There are many challenges that we confront today, including global terrorism, civil wars in the Middle East and Africa, poverty, starvation, and a torrent of natural disasters. Today we need more than slogans and symbols. There is a need for a real hope, a tangible hope that can be described, quantified, and transferred to those most in need.

A global “hope shortage” is particularly devastating for the young. Hope is about the future, and the young, whose lives are primarily in front of them, cannot prosper without trust and faith in what lies ahead. We know that even the healthiest adolescents struggle with issues of control and responsibility, love and relationships, problem–solving and coping, meaning and faith. For those youngsters who are poor, orphaned, abused, neglected, or handicapped, these developmental tasks will be even more difficult to accomplish.

In the past, there was little hope to be found in education, psychology, or counseling. Hope was for the philosophers and the priests. Fortunately, this neglect of hope is starting to change. Approximately a decade ago, I started to develop a broad, integrative theory of hope. As a clinical Psychologist, I could not believe that such an important human virtue had been neglected by my field. I gathered up the best ideas I could find from across different disciplines, including psychology, philosophy, theology, sociology, and even literary classics. I found that hope was invariably associated with one or more of the following human needs: *attachment, survival, mastery, or spirituality.* (Notice the overlap between the development tasks in the previous paragraph and these four components of hope.) In *Hope in the Age of Anxiety*, Dr. Henry Biler and I offered this definition of hopefulness (a.k.a. “fundamental hope”).

*Fundamental hope is a future-directed, personal four-channel emotion network, constructed from biological, psychological, and social resources. The four constituent channels are the mastery, attachment, survival, and spiritual systems (or sub-networks). The hope network regulates these systems via both feed-forward (expansion or growth) and feedback processes (maintenance or self-regulation) that generate a greater perceived probability of power and presence as well as protection and liberation* (Scioli & Biller, 2009).

In simpler terms, I view hope as a foundation that continues to evolve over the lifespan. Picture a five-story building. The five levels are developmental layers added over time. You enter life with basic needs for attachment, survival, mastery, and spirituality (Level 1). From birth, you enter into a complex set of interactions with the environment (Level 2). Your personality (character) traits emerge from this interplay of nature and nurture (Level 3). Deeply held beliefs and relationships may become centers of faith (Level 4). The inward (consciousness) and outward (behavior) manifestations of these four lower layers are your hopeful thoughts and actions.

Levels of Fundamental Hope: Biological, Social, and Psychological Factors

**Level 2: Nurture** via family, community, culture, worldviews

**Level 1: Nature** as reflected in wired capacities & needs

**Level 4: Faith** **System** reflecting “centers of value”

**Level 5: Expression of Hopefulness** as reflected in beliefs, images, feelings, action tendencies

**Level 3: Core Hope Traits** derived from nature & nurture

| *Beliefs*  I can Trust    *Feelings*  Connected *and*  Open  *Tendencies*  Care Recruitment | *Beliefs*  I can Cope  *Feelings*  Unfettered  *Tendencies*  Reality Negotiation | *Beliefs*  I have Strength    *Feelings*  Strong and Directed  *Tendencies*  Goal  Engagement | *Beliefs*  Universe is Kind  *Feelings*  Self-Transcendence  *Tendencies*  Dwelling in, and Seeking the  Sacred |
| --- | --- | --- | --- |
| *Faith in Self as Group Member*  Faith in Family, Friends, and Community | *Faith in Self as Coper*  Faith in Legal System, Govt.,  Medicine | *Faith in Self as an Effective Agent*  Faith in Work, Endeavors in Science or Art | *A Self Related to the Sacred*  Faith in a Higher Power, God (s), Nature, Cosmos |
| Trusting/Open | Resilient | Purposive | Centered |
| Reliable Contact | Adequate Care,  Co-Regulation | Mirroring Talent,  Supply of Ideals,  Sanctioned Goals | Spiritual  Experiences &  Opportunities |
| **Attachment**  **System** | **Survival**  **System** | **Mastery**  **System** | **Spiritual**  **Capacities**  **of Humanity** |

Hope is a state, trait, and a process. As a state, hope is more than a passing sensation. It is an energizing, organizing set of thoughts, feelings and behaviors (processes) that arise when we experience separation (attachment), threat (survival), challenge (mastery), or questions of meaning and ultimate value (spirituality). As a trait, hope is a virtue, a strength or character trait. Philosophers have labeled this second, trait-form “fundamental hope”.

With this explicit definition, it is possible to develop tools geared toward measuring and instilling “hope”. We can go beyond slogans, symbols, and indirect approaches that carry the “hope of hope” to actually working directly with the essence of hope itself. All of the workshop contents including the pre- and post- hope tests derive from this integrative theory of hope.
